# Single-belt vs. split-belt treadmill symmetry training: is there a perfect choice for gait rehabilitation post-stroke?

**DOI:** 10.3389/fphys.2024.1409304

**Published:** 2024-07-24

**Authors:** Chen Yang, Nicole Veit, Kelly McKenzie, Shreya Aalla, Kyle Embry, Ameen Kishta, Elliot Roth, Arun Jayaraman

**Affiliations:** ^1^ Shirley Ryan AbilityLab, Chicago, IL, United States; ^2^ Department of Physical Medicine and Rehabilitation, Feinberg School of Medicine, Northwestern University, Chicago, IL, United States; ^3^ Biomedical Engineering Department, McCormick School of Engineering, Northwestern University, Evanston, IL, United States

**Keywords:** treadmill, split-belt treadmill, gait adaptation, stroke, symmetry

## Abstract

Post-stroke gait asymmetry leads to inefficient gait and a higher fall risk, often causing limited home and community ambulation. Two types of treadmills are typically used for training focused on symmetry: split-belt and single belt treadmills, but there is no consensus on which treadmill is superior to improve gait symmetry in individuals with stroke. To comprehensively determine which intervention is superior, we considered multiple spatial and temporal gait parameters (step length, stride time, swing time, and stance time) and their symmetries. Ten individuals with stroke underwent a single session of split-belt treadmill training and single belt treadmill training on separate days. The changes in step length, stride time, swing time, stance time and their respective symmetries were compared to investigate which training improves both spatiotemporal gait parameters and symmetries immediately after the intervention and after 5 min of rest. Both types of treadmill training immediately increased gait velocity (0.08 m/s faster) and shorter step length (4.15 cm longer). However, split-belt treadmill training was more effective at improving step length symmetry (improved by 27.3%) without sacrificing gait velocity or step length. However, this step length symmetry effect diminished after a 5-min rest period. Split-belt treadmill training may have some advantages over single belt treadmill training, when targeting step length symmetry. Future research should focus on comparing the long-term effects of these two types of training and examining the duration of the observed effects to provide clinically applicable information.

## 1 Introduction

Stroke is a prevalent condition that affects 12.2 million people annually worldwide (Feigin et al., 2022). Over 80% of these individuals often experience deficits in walking ability, which impedes quality of life (Duncan et al., 2005; Tsao et al., 2022). Many gait rehabilitation approaches focus on improving gait velocity and spatiotemporal gait parameters, such as increasing step length or swing time, because changes in these metrics could lead to significant improvements in community walking ability and lower limb motor control (Bijleveld-Uitman et al., 2013). However, simply increasing walking speed or changing spatiotemporal parameters of both legs does not necessarily result in a more symmetric walking pattern. The person may walk faster but with an asymmetric walking pattern (Hsu et al., 2003; Wang et al., 2020). Gait asymmetry can be attributed to motor impairments such as impaired proprioception, decreased muscle strength, spasticity, and impaired balance control (Balasubramanian et al., 2007; Patterson et al., 2008; Titianova et al., 2008). The high prevalence of gait asymmetry after stroke has been linked to a higher risk of multiple negative consequences including impaired balance (Hendrickson et al., 2014), inefficient and increased metabolic costs (Ellis et al., 2013), and long-term musculoskeletal dysfunction (Jørgensen et al., 2000). Therefore, achieving spatiotemporal symmetry in gait is also crucial, as it facilitates inter-limb coordination, improves efficiency, and reduces the risk of falls (Wonsetler and Bowden, 2017).

To target gait symmetry in rehabilitation, the most common interventions are split-belt treadmill training and single-belt treadmill training, combined with visual and/or audio cueing. Split-belt treadmill training involves walking on a treadmill with belts moving at different speeds, which increases the stance time on the slow belt and swing time on the fast belt. This creates a motor error that reorganizes gait patterns, which are then applied when returning to tied-belt or overground walking (Hinton et al., 2020). Multiple studies have demonstrated that a single session of split-belt treadmill training instantly improves step length symmetry (Reisman et al., 2009; Lauzière et al., 2014; Malone and Bastian, 2014; Hirata et al., 2019). Similarly, studies have shown that combining single-belt treadmill training with visual or auditory cueing can improve gait symmetry in individuals post-stroke (Roerdink et al., 2007; Mainka et al., 2018; Shin and Chung, 2022). The visual or auditory cueing provides closed-loop sensory feedback, enabling real-time adjustments and improving the effectiveness of the training (Baram, 2013). While both interventions effectively improve gait symmetry, they also pose unique concerns. Reisman et al. (2013) observed that 12 training sessions of split-belt treadmill training improved only step length symmetry, but not temporal symmetries or gait velocity. Absence of control groups in prior split-belt studies further limits the evidence for the effectiveness of this training method. Although single-belt training is much more accessible and can successfully increase gait velocity, it does not enhance symmetry, unless combined with therapist cueing. Moreover, the evidence for using a single-belt combined with cueing to improve symmetry is far less established than split-belt treadmill training (Roerdink et al., 2007; Mainka et al., 2018; Shin and Chung, 2022). However, to date, there is no comparative analysis between split-belt and single-belt training combined with cueing interventions to establish which intervention is more effective for improving gait velocity, spatiotemporal parameters, and symmetry.

Therefore, this study aimed to 1) compare the acute effects of both interventions on spatiotemporal parameters and symmetry immediately after a single session of training and 2) determine whether the changes persisted after a 5-min period of seated rest, and 3) assess the amounts of symmetry improvement relative to the baseline symmetry for each treadmill training type. We hypothesized that both interventions would impact spatiotemporal parameters, but that split-belt training would improve step length symmetry more. Additionally, we predicted the 5-min rest period would reduce training effects since the newly-adopted movement pattern might diminish without reinforcement.

## 2 Materials and methods

### 2.1 Subjects

Ten participants with chronic stroke were recruited (Table 1). Inclusion criteria for the participants were: 1) history of a single, unilateral, supratentorial, stroke at least 1 year prior to participation 2) comfortable gait speed less than 1.0 m/s, and 3) medically stable with medical clearance to participate (absence of concurrent illness, including unhealed bone fractures or pressure sores, active injuries or infections, cardiopulmonary disease, osteoporosis, peripheral nerve damage in the lower limbs, and a history of any neurologic conditions). Exclusion criteria were as follows: 1) history of multiple strokes or bilateral strokes, 2) pregnant or nursing, 3) Modified Ashworth Score of three or greater in the lower extremity muscle groups, 4) Botox injections in the lower extremity within the last 4 months, or 5) presence of severe contractures in the lower extremities. All participants gave informed consent before participation. All participants gave informed consent before participation. All study-related procedures were approved by the Northwestern University Institutional Review Board (STU00215009), Northwestern University, Chicago, IL, United States. The study protocol was registered at clinicaltrials.gov (NCT05167786).

**TABLE 1:** Demographic and clinical characteristics of participants.

Subject	Sex	Age (years)	Height (cm)	Weight (kg)	Time since stroke (years)	Ankle-foot orthosis	Comfortable walking speed (m/s)	Fast walking speed (m/s)
S1	F	58	162.6	85.7	4.8	L articulated	0.84	1.13
S2	M	55	175.3	117.9	8.8	L carbon fiber	0.97	1.42
S3	M	69	172.7	77.1	7.5	L articulated	0.48	0.55
S4	M	61	172.7	77.1	4.8	R articulated	0.73	0.88
S5	F	49	157.5	99.8	6.5	None	0.91	1.26
S6	M	55	165.1	72.6	7.6	None	0.88	1.14
S7	M	57	175.3	70.3	8.6	R solid	0.73	0.90
S8	M	53	175.3	79.4	2.2	None	0.90	1.19
S9	F	62	160.0	72.6	7.6	None	0.93	1.19
S10	F	73	162.6	65.8	2.7	None	0.73	0.82
Mean (SD)	4F 6M	59.2 (6.9)	167.9 (6.7)	81.8 (15.0)	6.1 (2.2)	NA	0.8 (0.1)	1.0 (0.2)

### 2.2 Protocol

**FIGURE 1:**
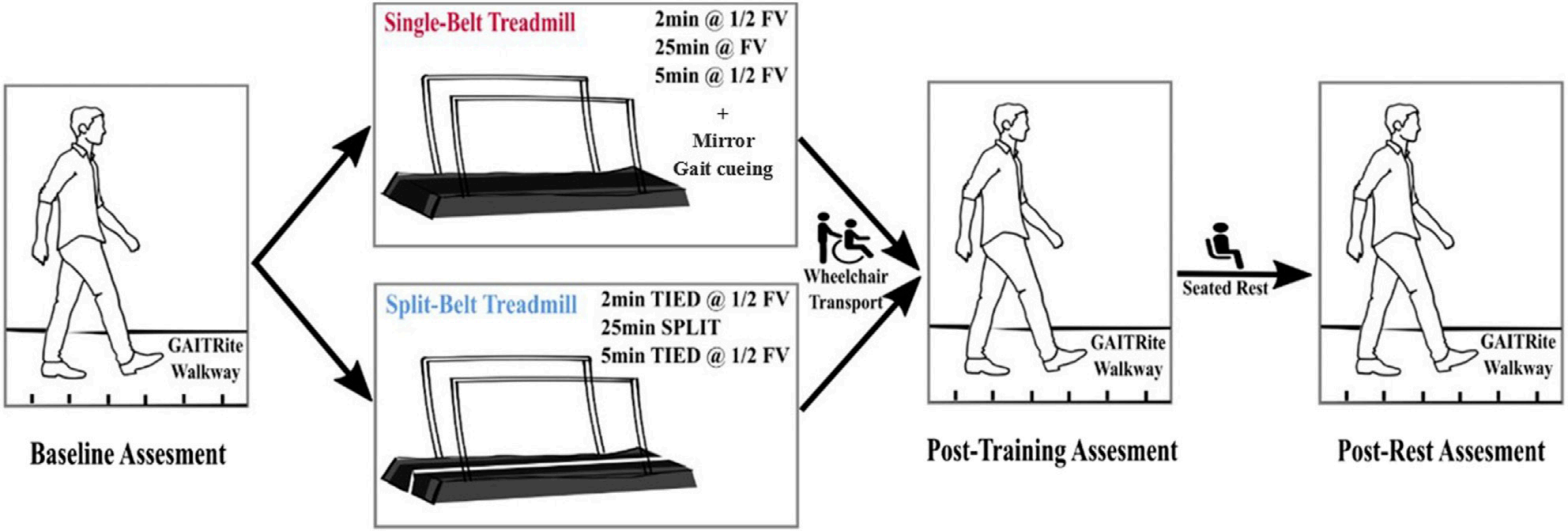
Schematic protocol of the study. During the split-belt walking, “TIED” or “SPLIT” indicate when two belts moved at the same speed or different speeds, respectively. The leg with the shorter step length was on the fast belt at FV, the leg with the longer step length was on the slow belt at ½FV.

Participants completed two interventions on separate days in a random order: one single-belt treadmill (Woodway^®^ United States, Waukesha, WI) session and one split-belt treadmill (Woodway^®^ United States, Waukesha, WI) session. Interventions started with a baseline gait assessment followed by 30-min of treadmill training. Participants were reassessed immediately after training and again after 5 min of seated recovery (Figure 1). Assessments included three 10-m walk tests at self-selected (SSV) and three 10-m walk test at fast velocity (FV) over a GAITRite^®^ walkway (CIR Systems Inc., NJ, United States). For FV, participants were instructed to walk as fast and safely as possible. Baseline trials were processed to 1) calculate average gait velocities and 2) determine which leg had a shorter step length.

The split-belt treadmill (Woodway^®^ United States, Waukesha, WI) training started with 2 min of tied-belt walking (both belts at ½ FV), 25 min of split-belt walking (fast belt at FV, slow belt at ½FV), and finally 5 min of tied-belt walking (goal: both belts at ½ FV). The leg with shorter step length was placed on the fast belt. Single-belt treadmill training (Woodway^®^ United States, Waukesha, WI) consisted of 2 min warm up (½ fast over-ground speed), 25 min of training (fast over-ground speed), and 5 min of cool down (½ fast over-ground speed). During single-belt training, a physical therapist cued for spatiotemporal symmetry and a mirror was placed in front of the treadmill for visual feedback. Before the first split-belt training session, each participant underwent a familiarization period on the treadmill to get used to walking on the device. The participants were encouraged not to hold on to the handrails during both sessions. A ceiling-mounted safety harness was utilized without providing body weight support. Sitting breaks were provided upon participant’s request. Blood pressure (BP) was assessed pre and post ambulation. Heart rate (HR) and Rated Perceived Exertion (RPE) were monitored throughout the training. Participants were transported *via* wheelchair to complete post-training assessments to avoid walking between training and assessment.

### 2.3 Data collection and analysis

During each assessment, step length, stride length, swing time, and stance time of over-ground gait were collected by the GAITRite walkway. Spatiotemporal symmetry indices were calculated (Eq. 1) for each participant from the obtained metrics.

$$Symmetry\;Index=\frac{\left|X_{shorter}\;-{\;X}_{longer}\right|}{0.5\left(X_{shorter}+X_{longer}\right)}$$ (1)

$X_{shorter}$ and $X_{longer}$ are the value of each spatiotemporal parameter on the shorter and longer side, respectively. The spatiotemporal parameters and symmetry values were calculated for SSV at baseline, post-training (immediate change), and post 5 min of rest (delayed change). A smaller symmetry index value indicates a more symmetric gait. Spatiotemporal symmetries were used to obtain the percent changes from baseline as shown in Eqs 2, 3. A positive percent change indicates a greater post value compared to baseline or increased asymmetry.

$$Immediate\;change=\frac{\left(V_{Post-training}-V_{Baseline}\right)}{V_{Baseline}}\times 100\%$$ (2)

$$Delayed\;change=\frac{\left(V_{Post-rest}-V_{Baseline}\right)}{V_{Baseline}}\times 100\%$$ (3)

### 2.4 Statistical analysis

The Kolmogorov–Smirnov test was used to assess the normality of the data. The data of all the outcomes was normally distributed. Generalized estimating equations (GEEs; Link Function = Identity; Structure of Covariance Matrix = Exchangeable) were conducted to test the effects of treadmill intervention (single vs. split belt) and time (baseline vs. post-training vs. post-rest) on all gait spatiotemporal and symmetry parameters. An alpha level was set at 0.05 *a priori*. Interaction effects were examined by *post hoc* pairwise comparisons with sequential Bonferroni adjustments. GEE was selected because it obtains higher power with a small sample size compared to repeated measured analysis of variance (Ma et al., 2012; Naseri et al., 2016). Spearman correlations were calculated to test the relationship between the symmetry changes (Immediate/Delayed change) and baseline gait parameters. Statistics were performed in SPSS (SPSS Statistics v27, IBM Corp., US).

## 3 Results

### 3.1 Spatiotemporal gait parameters

Statistical results are in Table 2. We found significant changes with time for gait velocity (X^2^ = 21.45, *p* < 0.001), shorter (X^2^ = 4.55, *p* = 0.033) and longer (X^2^ = 13.27, *p* = 0.001) step lengths, shorter (X^2^ = 12.76, *p* = 0.002) and longer (X^2^ = 12.87, *p* = 0.002) stride lengths, and shorter stance time (X^2^ = 9.03, *p* = 0.011). Pairwise comparisons showed gait velocity improved (10.13% increase, *p* < 0.001) after rest compared to baseline for both training types. In both training types, shorter step length increased immediately after training (6.41% increase, *p* = 0.004). Longer step length (5.32% increase, *p* = 0.004), shorter stride length (6.96% increase, *p* = 0.001) and longer stride length (7.14% increase, *p* = 0.001) all increased after rest compared to baseline. Shorter stance time (3.61% decrease, *p* = 0.044) became even shorter after rest.

**TABLE 2:** Gait parameters in self-selected velocity. Values are shown as mean ± standard error in the table. * indicates significant difference.

	Single-belt treadmill			Split-belt treadmill			*p*-value		
	Baseline	Post-training	Post-rest	Baseline	Post-training	Post-rest	Treadmill effect	Time effect	Treadmill * time interaction
Spatiotemporal parameters									
Gait velocity (m/s)	0.78 ± 0.07	0.82 ± 0.08	0.84 ± 0.06	0.80 ± 0.06	0.82 ± 0.07	0.89 ± 0.07	0.218	*<0.001	0.271
Longer step length (cm)	55.06 ± 3.35	57.58 ± 4.22	59.03 ± 3.36	57.77 ± 3.82	57.39 ± 3.95	59.78 ± 3.69	0.300	*0.001	0.103
Shorter step length (cm)	44.39 ± 3.38	47.24 ± 3.47	49.06 ± 2.91	46.68 ± 3.45	49.66 ± 3.75	50.31 ± 3.35	*0.033	*0.002	0.502
Longer stride length (cm)	99.91 ± 6.22	105.28 ± 7.53	108.63 ± 6.07	104.73 ± 7.02	107.65 ± 7.61	110.63 ± 6.79	0.113	*0.002	0.557
Shorter stride length (cm)	99.11 ± 6.15	104.67 ± 7.50	107.67 ± 5.97	103.99 ± 6.96	106.96 ± 7.58	109.57 ± 6.69	0.103	*0.002	0.563
Longer stance time (s)	0.99 ± 0.07	0.99 ± 0.09	0.95 ± 0.07	0.98 ± 0.08	0.97 ± 0.09	0.94 ± 0.07	0.234	*0.027	0.913
Shorter stance time (s)	0.84 ± 0.06	0.85 ± 0.08	0.80 ± 0.06	0.83 ± 0.07	0.82 ± 0.08	0.79 ± 0.06	0.248	*0.011	0.797
Longer swing time (s)	0.51 ± 0.03	0.49 ± 0.03	0.50 ± 0.03	0.50 ± 0.03	0.50 ± 0.02	0.50 ± 0.02	0.765	0.193	0.371
Shorter swing time (s)	0.35 ± 0.01	0.35 ± 0.01	0.36 ± 0.01	0.35 ± 0.01	0.35 ± 0.01	0.35 ± 0.01	0.359	0.839	0.943
Symmetry parameters									
Step length symmetry	0.23 ± 0.06	0.21 ± 0.03	0.19 ± 0.03	0.22 ± 0.04	0.16 ± 0.02	0.18 ± 0.03	*<0.001	0.069	*0.005
Stride length symmetry	0.03 ± 0.01	0.03 ± 0.01	0.03 ± 0.01	0.03 ± 0.01	0.04 ± 0.01	0.03 ± 0.01	0.623	0.167	0.484
Stance time symmetry	0.17 ± 0.02	0.16 ± 0.02	0.17 ± 0.02	0.17 ± 0.02	0.17 ± 0.02	0.17 ± 0.02	0.548	0.981	0.542
Swing time symmetry	0.35 ± 0.04	0.33 ± 0.05	0.33 ± 0.03	0.35 ± 0.04	0.35 ± 0.03	0.33 ± 0.03)	0.760	0.545	0.552

### 3.2 Symmetry

**FIGURE 2:**
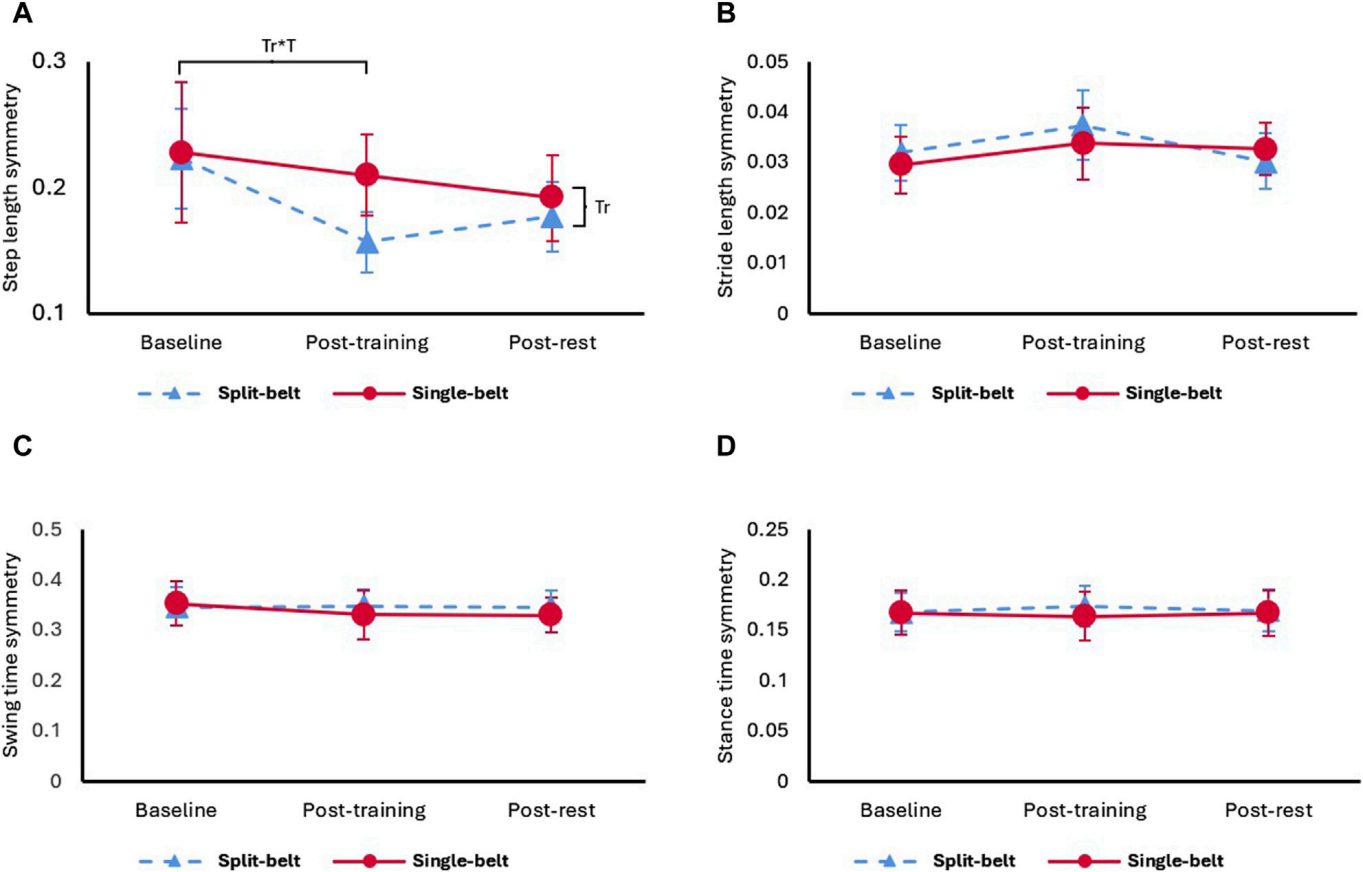
**(A)** Step length symmetry, **(B)** stride length symmetry, **(C)** swing time symmetry, and **(D)** stance time symmetry under self-selected velocity in two treadmill training. Tr: main effect of treadmill, Tr*T: interaction effect of treadmill and time.

Only step length symmetry showed a significant treadmill*time interaction (X^2^ = 10.51, *p* = 0.005) and a significant treadmill effect (X^2^ = 11.40, *p* < 0.001), all other symmetry parameters did not change with time or treadmill. Figure 2 shows step length symmetry immediately improved only after split-belt (27.27% decrease, *p* = 0.040), but not single-belt treadmill training (*p* = 0.509). However, after rest, there was no significant difference between the two treadmill training types.

### 3.3 Correlations

**FIGURE 3:**
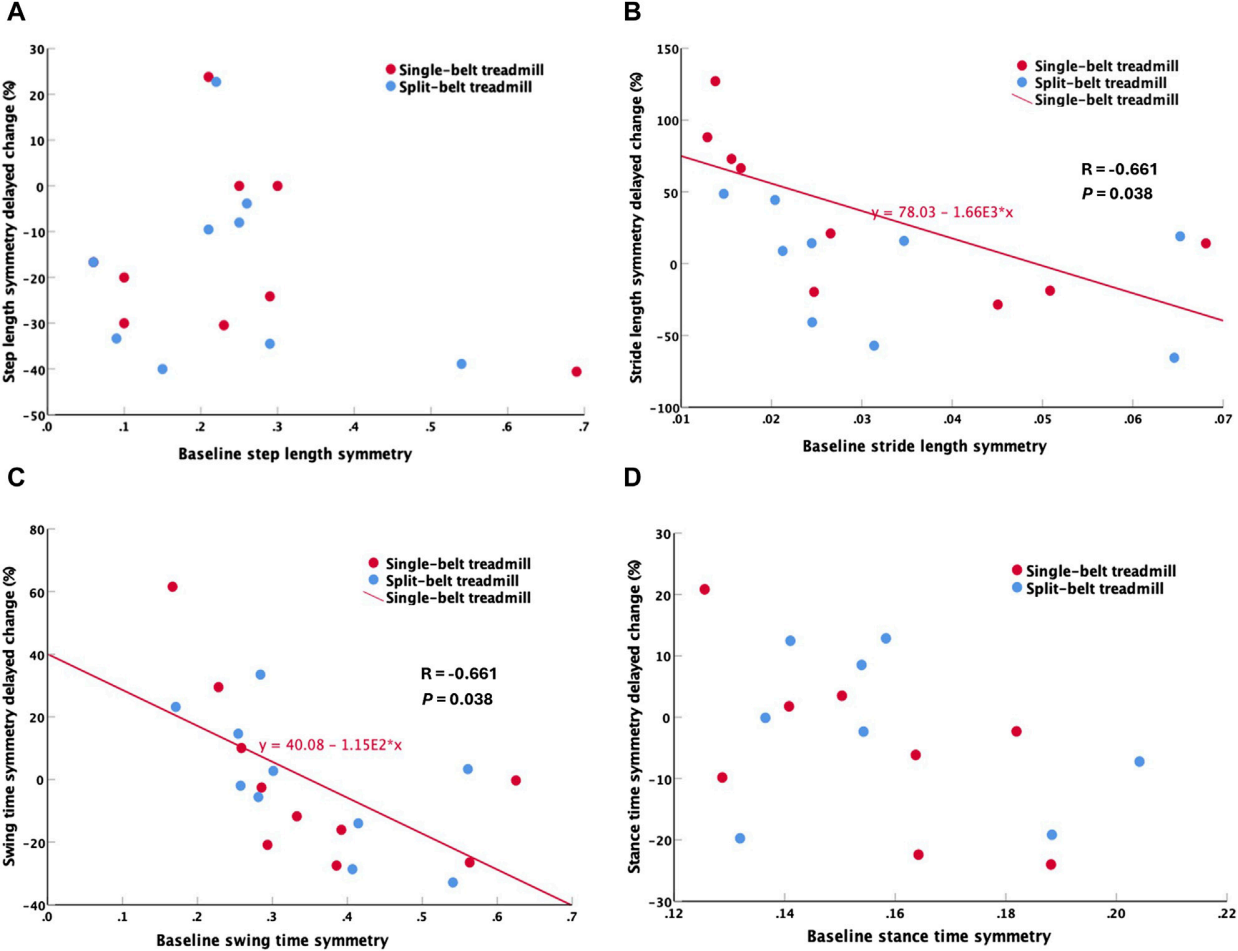
Correlations between baseline and delayed change. **(A)** Step length symmetry, **(B)** Stride length symmetry, **(C)** Swing time symmetry, **(D)** Stance time symmetry. Stride length symmetry and swing time symmetry showed significant correlations between baseline and delayed change only in single-belt treadmill training.

The immediate change of symmetry was not statistically correlated with any baseline gait parameters for either treadmill training type. However, the delayed change of SSV stride length symmetry (R = −0.661, *p* = 0.038; Figure 3B) and swing time symmetry (R = −0.661, *p* = 0.038; Figure 3C) were significantly correlated with baseline symmetry in single-belt treadmill training. Greater spatiotemporal symmetry improvements were associated with worse spatiotemporal symmetry at baseline for the single-belt treadmill only.

## 4 Discussion

This is the first comparison of single-session effects of single-belt and split-belt treadmill training on spatiotemporal measures in stroke survivors. Our results demonstrated that: 1) Both treadmill training types immediately increased shorter step length. Step length symmetry immediately improved significantly only after split-belt treadmill training, without compromising temporal symmetry or gait velocity. 2) Post-training rest of 5 min improved gait velocity and spatial gait performance. However, unlike the gait velocity and spatial gait performance, step length symmetry was insignificantly different from baseline after the rest. 3) Swing time and stride length symmetry improvements were associated with poor baseline levels in single-belt but not associated with baseline levels in split-belt training. These findings suggest that split-belt treadmill training might be superior to single-belt treadmill training when specifically targeting step length symmetry. However, both types of training were found to improve gait velocity and shorter step length. More research is needed to compare the long-term effects since the step length symmetry effects diminish after 5 min of rest.

### 4.1 Immediate training effects

Shorter step length was the only spatiotemporal parameter to show immediate training effects. The shorter step length immediately increased after both treadmill training, as observed in previous studies (Gama et al., 2017; Betschart et al., 2018). This could be attributed to the increased range of motion of the limb at faster velocities. Our split-belt training protocol placed the shorter step length side on the fast belt, which increased the step length on that side while maintaining the longer step length on the slow belt, leading to immediate improvement in symmetry. In contrast, with single-belt training, step length increased on both sides, thus not altering step length symmetry.

### 4.2 Rest effects

We hypothesized that the training effects on spatiotemporal gait parameters would diminish after 5 min of rest, but our results showed the opposite. The rest reduced fatigue, which possibly amplified the training effect. Reisman et al. (2013) reported no gait velocity improvement after 12 split-belt training sessions. This might also be attributed to fatigue that was induced by their longer training duration at each session. Stroke survivors adapt to split-belt training slower than neurologically intact controls do (Savin et al., 2013; Tyrell et al., 2014). To develop an effective split-belt training paradigm, future studies could test various training and rest durations.

### 4.3 Differences between treadmills

In this study, only split-belt treadmill training immediately improved step length symmetry without compromising gait velocity or other symmetry parameters. Additionally, single-belt treadmill baseline values were negatively correlated with the spatiotemporal symmetry improvement. Therefore, single-belt treadmill training requires worse baseline symmetry in stride length and swing time to generate spatiotemporal symmetry improvements, while this was not necessary for split-belt treadmill training. This supports choosing split-belt treadmill training to target symmetry in stroke rehabilitation. Using the error augmentation strategy (Reisman et al., 2013), we placed the leg with shorter step length on the fast belt, the fast belt further shortened the step length of the leg, exaggerating the “error” of step length asymmetry. Afterwards, when assessing the effects on gait, the aftereffects led to participants correcting “the error”, resulting in the observed increased step length in the short side and maintenance of step length in longer side which in turn improved step length symmetry (Helm and Reisman, 2015). Split-belt training induces proprioceptive feedback through walking in an abnormal pattern, which informs the central pattern generators and supraspinal centers to modify the motor output to adapt and achieve a new gait pattern (Hinton et al., 2020). In contrast, single-belt treadmill walking maintains a pattern similar to over ground walking, and the participants correct the walking pattern according to the visual or auditory feedback (Pereira et al., 2016), where we observe an increase on step length symmetry by increasing both the short and long step lengths, although the improvements on symmetry are not significant.

### 4.4 Clinical implications and future research directions

Our study suggests that both single-belt and split-belt treadmill training effectively improve gait speed and step length on the shorter side in individuals with asymmetrical gait patterns. More interestingly, temporal symmetry remained unchanged after split-belt treadmill training. Our results indicate that split-belt treadmill training improves step length symmetry without compromising temporal symmetry, aligning with findings from a previous study by Lewek et al. (2018). Clinicians should incorporate split-belt treadmill training to target step length symmetry and consider additional strategies to maintain these improvements. This study is limited by its small sample size and its use of single training session results. To provide better suggestions for clinicians, future studies could consider increasing the sample size and conducting multiple sessions of training. Long-term effects of both training types should be investigated to understand the sustainability of improvements.

## 5 Conclusion

Our results demonstrate that both single-belt and split-belt treadmill training equally improve gait speed and step length on the shorter side. Split-belt training resulted in a significant improvement in step length symmetry immediately after training without impairing other temporal symmetries. However, this effect diminished after a 5-min rest. Interestingly, the short period of post-training rest reinforced spatial gait improvements from both types of treadmill training, which might be a result of reduced fatigue. However, further studies are needed to explore the long-term training effects between different types of treadmill, as the step length symmetry tends to converge between two treadmill training after a 5-min rest period. These findings highlight the potential of split-belt treadmill training to enhance gait symmetry in stroke rehabilitation. By refining and extending these training protocols, we have the opportunity to significantly improve patient outcomes, leading to more efficient and safer ambulation for individuals post-stroke.

## Data Availability

The raw data supporting the conclusion of this article will be made available by the authors, without undue reservation.

## References

[B1] Balasubramanian C. K. Bowden M. G. Neptune R. R. Kautz S. A. (2007). Relationship between step length asymmetry and walking performance in subjects with chronic hemiparesis. Archives Phys. Med. Rehabilitation 88, 43–49. 10.1016/j.apmr.2006.10.004 17207674

[B2] Baram Y. (2013). Virtual sensory feedback for gait improvement in neurological patients. Front. Neurology 4, 138. 10.3389/fneur.2013.00138 PMC3796285 24133478

[B3] Betschart M. Mcfadyen B. J. Nadeau S. (2018). Repeated split-belt treadmill walking improved gait ability in individuals with chronic stroke: a pilot study. Physiother. Theory Pract. 34, 81–90. 10.1080/09593985.2017.1375055 28901824

[B4] Bijleveld-Uitman M. Van De Port I. Kwakkel G. (2013). Is gait speed or walking distance a better predictor for community walking after stroke? J. Rehabil. Med. 45, 535–540. 10.2340/16501977-1147 23584080

[B5] Duncan P. W. Zorowitz R. Bates B. Choi J. Y. Glasberg J. J. Graham G. D. (2005). Management of adult stroke rehabilitation care: a clinical practice guideline. Stroke 36, e100–e143. 10.1161/01.STR.0000180861.54180.FF 16120836

[B6] Ellis R. G. Howard K. C. Kram R. (2013). The metabolic and mechanical costs of step time asymmetry in walking. Proc. R. Soc. B Biol. Sci. 280, 20122784. 10.1098/rspb.2012.2784 PMC3574372 23407831

[B7] Feigin V. L. Brainin M. Norrving B. Martins S. Sacco R. L. Hacke W. (2022). World stroke organization (WSO): global stroke fact sheet 2022. Int. J. Stroke 17, 18–29. 10.1177/17474930211065917 34986727

[B8] Gama G. L. Celestino M. L. Barela J. A. Forrester L. Whitall J. Barela A. M. (2017). Effects of gait training with body weight support on a treadmill versus overground in individuals with stroke. Archives Phys. Med. rehabilitation 98, 738–745. 10.1016/j.apmr.2016.11.022 28034719

[B9] Helm E. E. Reisman D. S. (2015). The split-belt walking paradigm exploring motor learning and spatiotemporal asymmetry poststroke. Phys. Med. Rehabilitation Clin. N. Am. 26, 703–713. 10.1016/j.pmr.2015.06.010 PMC4631066 26522907

[B10] Hendrickson J. Patterson K. K. Inness E. L. Mcilroy W. E. Mansfield A. (2014). Relationship between asymmetry of quiet standing balance control and walking post-stroke. Gait Posture 39, 177–181. 10.1016/j.gaitpost.2013.06.022 23877032

[B11] Hinton D. C. Conradsson D. M. Paquette C. (2020). Understanding human neural control of short-term gait adaptation to the split-belt treadmill. Neuroscience 451, 36–50. 10.1016/j.neuroscience.2020.09.055 33039522

[B12] Hirata K. Hanawa H. Miyazawa T. Kubota K. Sonoo M. Kokubun T. (2019). Adaptive changes in foot placement for split-belt treadmill walking in individuals with stroke. J. Electromyogr. Kinesiol. 48, 112–120. 10.1016/j.jelekin.2019.07.003 31325672

[B13] Hsu A. L. Tang P. F. Jan M. H. (2003). Analysis of impairments influencing gait velocity and asymmetry of hemiplegic patients after mild to moderate stroke. Arch. Phys. Med. Rehabil. 84, 1185–1193. 10.1016/s0003-9993(03)00030-3 12917858

[B14] Jørgensen L. Crabtree N. Reeve J. Jacobsen B. (2000). Ambulatory level and asymmetrical weight bearing after stroke affects bone loss in the upper and lower part of the femoral neck differently: bone adaptation after decreased mechanical loading. Bone 27, 701–707. 10.1016/s8756-3282(00)00374-4 11062359

[B15] Lauzière S. Mieville C. Betschart M. Duclos C. Aissaoui R. Nadeau S. (2014). Plantarflexion moment is a contributor to step length after-effect following walking on a split-belt treadmill in individuals with stroke and healthy individuals. J. rehabilitation Med. 46, 849–857. 10.2340/16501977-1845 25074249

[B16] Lewek M. D. Braun C. H. Wutzke C. Giuliani C. (2018). The role of movement errors in modifying spatiotemporal gait asymmetry post stroke: a randomized controlled trial. Clin. Rehabil. 32, 161–172. 10.1177/0269215517723056 28750549 PMC5748372

[B17] Mainka S. Wissel J. Völler H. Evers S. (2018). The use of rhythmic auditory stimulation to optimize treadmill training for stroke patients: a randomized controlled trial. Front. neurology 9, 755. 10.3389/fneur.2018.00755 PMC6149244 30271375

[B18] Malone L. A. Bastian A. J. (2014). Spatial and temporal asymmetries in gait predict split-belt adaptation behavior in stroke. Neurorehabilitation neural repair 28, 230–240. 10.1177/1545968313505912 24243917 PMC4336782

[B19] Ma Y. Mazumdar M. Memtsoudis S. G. (2012). Beyond repeated-measures analysis of variance: advanced statistical methods for the analysis of longitudinal data in anesthesia research. Regional Anesth. Pain Med. 37, 99–105. 10.1097/AAP.0b013e31823ebc74 PMC3249227 22189576

[B20] Naseri P. Majd H. A. Kariman N. Sourtiji A. (2016). Comparison of generalized estimating equations (GEE), mixed effects models (MEM) and repeated measures ANOVA in analysis of menorrhagia data. Archives Adv. Biosci. 7, 32–40.

[B21] Patterson K. K. Parafianowicz I. Danells C. J. Closson V. Verrier M. C. Staines W. R. (2008). Gait asymmetry in community-ambulating stroke survivors. Arch. Phys. Med. Rehabil. 89, 304–310. 10.1016/j.apmr.2007.08.142 18226655

[B22] Pereira M. P. Gobbi L. T. Almeida Q. J. (2016). Freezing of gait in Parkinson’s disease: evidence of sensory rather than attentional mechanisms through muscle vibration. Park. Relat. Disord. 29, 78–82. 10.1016/j.parkreldis.2016.05.021 27245918

[B23] Reisman D. S. Mclean H. Keller J. Danks K. A. Bastian A. J. (2013). Repeated split-belt treadmill training improves poststroke step length asymmetry. Neurorehabilitation Neural Repair 27, 460–468. 10.1177/1545968312474118 23392918 PMC3738184

[B24] Reisman D. S. Wityk R. Silver K. Bastian A. J. (2009). Split-belt treadmill adaptation transfers to overground walking in persons poststroke. Neurorehabilitation Neural Repair 23, 735–744. 10.1177/1545968309332880 19307434 PMC2811047

[B25] Roerdink M. Lamoth C. J. Kwakkel G. Van Wieringen P. C. Beek P. J. (2007). Gait coordination after stroke: benefits of acoustically paced treadmill walking. Phys. Ther. 87, 1009–1022. 10.2522/ptj.20050394 17553922

[B26] Savin D. N. Tseng S. C. Whitall J. Morton S. M. (2013). Poststroke hemiparesis impairs the rate but not magnitude of adaptation of spatial and temporal locomotor features. Neurorehabil Neural Repair 27, 24–34. 10.1177/1545968311434552 22367915 PMC5012177

[B27] Shin J. Chung Y. (2022). The effects of treadmill training with visual feedback and rhythmic auditory cue on gait and balance in chronic stroke patients: a randomized controlled trial. NeuroRehabilitation 51, 443–453. 10.3233/NRE-220099 35964207

[B28] Titianova E. B. Peurala S. H. Pitkänen K. Tarkka I. M. (2008). Gait reveals bilateral adaptation of motor control in patients with chronic unilateral stroke. Aging Clin. Exp. Res. 20, 131–138. 10.1007/BF03324759 18431080

[B29] Tsao C. W. Aday A. W. Almarzooq Z. I. Alonso A. Beaton A. Z. Bittencourt M. S. (2022). Heart disease and stroke statistics-2022 update: a report from the American heart association. Circulation 145, e153–e639. 10.1161/CIR.0000000000001052 35078371

[B30] Tyrell C. M. Helm E. Reisman D. S. (2014). Learning the spatial features of a locomotor task is slowed after stroke. J. Neurophysiol. 112, 480–489. 10.1152/jn.00486.2013 24790172 PMC4064415

[B31] Wang Y. Mukaino M. Ohtsuka K. Otaka Y. Tanikawa H. Matsuda F. (2020). Gait characteristics of post-stroke hemiparetic patients with different walking speeds. Int. J. Rehabilitation Res. 43, 69–75. 10.1097/MRR.0000000000000391 PMC7028468 31855899

[B32] Wonsetler E. C. Bowden M. G. (2017). A systematic review of mechanisms of gait speed change post-stroke. Part 1: spatiotemporal parameters and asymmetry ratios. Top. Stroke Rehabil. 24, 435–446. 10.1080/10749357.2017.1285746 28220715 PMC5875693

